# Risk factors for oral and esophageal candidiasis during budesonide treatment in eosinophilic esophagitis patients

**DOI:** 10.1007/s11739-026-04324-y

**Published:** 2026-04-30

**Authors:** Irene Spinelli, Maria Parmigiani, Francesca Fianchi, Arianna Aruanno, David Longhino, Federica Castri, Cristiano Caruso, Francesca Romana Ponziani, Maria Elena Riccioni, Gianluca Ianiro, Antonio Gasbarrini

**Affiliations:** 1 CEMAD, Fondazione Policlinico Universitario A. Gemelli IRCCS, Largo Agostino Gemelli, 8, 00168 Rome, Italy; 2 Fondazione Poliambulanza Istituto Ospedaliero, Via Leonida Bissolati, 57, 25124 Brescia, BS Italy; 3 UOSD Allergologia e Immunologia Clinica, Fondazione Policlinico Universitario A. Gemelli IRCCS, Largo Agostino Gemelli, 8, 00168 Rome, Italy; 4 Anatomia Patologica Generale, Fondazione Policlinico Universitario A. Gemelli IRCCS, Largo Agostino Gemelli, 8, 00168 Rome, Italy; 5 Endoscopia Digestiva Chirurgica, Fondazione Policlinico Universitario A. Gemelli IRCCS, Roma, Italy

**Keywords:** Eosinophilic esophagitis, Budesonide, Proton pump inhibitor

## Abstract

Eosinophilic esophagitis (EoE) first-line therapy is represented by budesonide orodispersible tablets (BOT), but a very low number of patients develop oral and esophageal candidiasis. The aim of the study is to evaluate risk factors for the development of oral and esophageal candidiasis during budesonide therapy in EoE patients. A retrospective study was conducted to include all EoE patients in BOT therapy referred to our center. Prevalence, localization, and time of presentation of candidiasis, eosinophilic count in gastroscopy before BOT therapy, and concomitant treatment with proton pump inhibitors (PPI) and its dosage were reviewed. A total of 46 EoE patients in BOT therapy were included in the study. 7 patients (15%) developed oral and/or esophageal candidiasis. 4 patients (57%) of this group were in concomitant PPI therapy, all at 40 mg per day or more dosage, whereas 38% of patients without mycosis were taking this medication (41% at 40 mg per day or more). The mean eosinophil count before treatment was 45/HPF in patients with candidiasis infection and 33/HPF for the others without. Concomitant PPI therapy and high eosinophilic count may represent risk factors in developing mycosis in EoE patients.

## Introduction

Eosinophilic esophagitis (EoE) is a chronic and progressive disease characterized by eosinophil-predominant inflammation of the esophagus. The etiology of the disease is multifactorial, comprising genetic, antigenic, environmental, and intrinsic immune system-related factors [1].

The estimated global prevalence in adults is approximately 42.2 per 100,000 inhabitants, with an observed increase in incidence over the past decades. An improved disease recognition offers only a partial explanation for this epidemiological escalation [2–4]. In order to make a diagnosis, multiple esophageal biopsies are required, with a minimum of 15 eosinophils per high-power field (HPF) being necessary for a positive result [5].

Therapeutic options include dietary and pharmacologic treatments.

Until 2017, first-line therapy was represented by proton pump inhibitors (PPI). Even if PPIs are all licensed for the treatment of reflux esophagitis and non-erosive reflux disease, a specific indication for EoE is not part of the prescribing information. Subsequently, the advent of topical corticosteroids marked a significant shift in the therapeutic landscape. Budesonide orodispersible tablet (BOT) is a corticosteroid formulation approved by the European Medicines Agency (EMA) for on-label use in EoE [6]. The approval was based on a randomized controlled trial (RCT) showing a clinical–histological remission rate of up to 57.6% after six weeks of treatment and of 85% after 12 weeks of treatment and based on an open-label induction study showing a clinical–histological remission rate of 69.9% after six weeks of treatment [7]. Its efficacy in terms of clinical, endoscopic, and histological improvement is nowadays well established. Topical steroids have also demonstrated a favorable safety profile for induction and maintenance of remission in the  long term [8, 9]. RCTs investigating different topical steroid preparations have, in general, demonstrated a favorable safety profile in the short and medium term, with no significant increase in the incidence of serious adverse events among patients receiving active drugs compared to those receiving placebo [7]. Esophageal candidiasis (EC) has been reported in up to 15% of patients undergoing topical steroid treatment of all kinds [10–17]. In such instances, the administration of antifungal agents is recommended as a means of resolving the fungal infection. Long-term RCTs and real-life prospective studies are required to evaluate the incidence and severity of potential adverse effects associated with all kinds of topical steroidal treatment and to establish a framework for follow-up strategies for patients undergoing long-term topical steroid therapy.

The objective of this study is to identify the risk factors associated with the development of oral and esophageal candidiasis during budesonide orodispersible therapy in patients with eosinophilic esophagitis. To date, no studies have addressed this issue yet.

## Material and methods

A retrospective study was conducted on all patients diagnosed with eosinophilic esophagitis who were undergoing the induction (1 mg twice a day) and the maintenance (0.5 mg twice a day) phase of BOT therapy at the Fondazione Policlinico Universitario A. Gemelli IRCCS between March 2022 and November 2023. The study included 46 patients, of whom 40 were male.

Patients were selected by querying the Rare Gastrointestinal and Liver Diseases database of the Department of Internal Medicine and Gastroenterology, and all cases taking BOT therapy for active eosinophilic esophagitis were included.

The diagnosis of pathology was histological with a peak eosinophil count ≥ 15 eosinophils per high-power field in at least one high-power field on esophageal biopsy, in the absence of other causes of esophageal eosinophilia.

Patients took the tablets by pressing the tip of the tongue against the roof of the mouth, twice a day, without eating, drinking, brushing teeth, or rinsing the mouth for at least 30 min after taking the tablets.

All types of adverse events were documented during the utilization of BOT. In the case of patients presenting with candidiasis, data were collected on the precise location of the infection. Oral candidiasis was reported by patients and subsequently confirmed by objective oral examination, manifesting as white or erythematous patches in the mouth and throat.  Follow-up endoscopies identified scattered plaque-like lesions adhering to the mucosa as a fungal infection of the esophagus, which was subsequently confirmed by histological analysis.

Characteristics of infection presentation were annotated in terms of time of onset after initiation of BOT (weeks), duration of infection (days),  presence of symptoms, and therapeutic strategies for treatment (topical or systemic antifungal therapy).

The data obtained from the gastroscopic examinations conducted prior to the administration of steroid therapy were subjected to analysis. For each examination, endoscopic features were quantified using the validated EoE Endoscopic Reference Score (EREFS).

The histological findings were evaluated by a detailed examination of the pathology reports, with specific attention paid to the precise location of the biopsy, the number of eosinophilic peaks, the presence of eosinophilic micro-abscesses, basal cell hyperplasia, and other relevant features.

Histological remission was defined as peak eosinophil density < 15 eosinophils per 0.3 mm^2^. Deep/complete remission was defined as peak eosinophil density < 5 eosinophils per 0.3 mm^2^ [18].

We looked at the same data for gastroscopy performed 12 weeks after the start of induction treatment.

Each case of discontinuation of BOT for disabling candidiasis was reviewed. Time and reason for discontinuation were analyzed.

In addition, concomitant therapies to BOT were subject to careful evaluation. The review encompassed the assessment of whether patients included in the study were concurrently taking other medications at the time of steroid assumption, with a particular focus on the use of PPI and their dosage. This study was approved by our Ethical Committee, as all patients were enrolled in an eosinophilic esophagitis registry previously approved by the Committee. 

### Statistical analysis

Categorical variables are expressed as counts and percentages, whereas continuous variables are expressed as means and standard deviations (SDs). The normality of data has been verified with the Kolmogorov-Smirnov test. Proportions were compared applying the Chi-square test. A p-value < 0.05 was considered statistically significant. All the statistical analyses have been performed with Statistical Package for the Social Sciences (SPSS) statistical software (Realeased 2017, IBM SPSS Statistics for Windows, Version 25.0; IBM Corp., Armonk, NY).

## Results

A total of 46 patients undergoing BOT therapy for EoE, comprising 40 males and 6 females, were included in the study.

The mean age of the patients was 37 ± 11 years (SD).

A comparison was made between two subgroups: one comprising 39 patients without candidiasis and one comprising seven patients with mycosis. The clinical and pathological features are presented in Table 1.

**Table 1:** Clinical and pathological features

	Total (*n* = 46)	Patients without candidiasis infection (*n* = 39)	Patients with candidiasis infection (*n* = 7)	*p*-value
Males	40	35	5	ns
Age years ± SDs	37 ± 11	36 ± 12	42 ± 9	ns
Peak eosinophil count/HPF before BOT therapy ± SDs	35 ± 20	33 ± 21	45 ± 8.6	ns
Previous EoE treatment	40 (87)	34 (87)	6 (86)	ns
PPI	40 (87)	34 (87)	6 (86)	ns
Swallowed topical steroids	21 (46)	18 (46)	3 (43)	ns
Concomitant PPI therapy	19 (41)	15 (38)	4 (57)	ns
No PPI concomitant therapy	27 (59)	24 (62)	3 (43)	ns
PPI < 40 mg	36 (78)	33 (85)	3 (43)	< 0.05
EREFS before BOT ≥ 2	26 (57)	22 (56)	4 (57)	ns
EREFS after BOT ≥ 2	18 (39)	15 (38)	3 (42)	ns

Values are expressed as *n* (%)

*SDs* standard deviations, *HPF* high power field, *BOT* budesonide orodispersible tablets, *EoE* eosinophilic esophagitis, *PPI* proton pump inhibitors, *EREFS* EoE Endoscopic Reference Score, *ns* not significant (*p*-value > 0.05)

### Candidiasis presentation and treatment

The sole adverse event documented during BOT therapy was oral/esophageal candidiasis. No other instances were reported.

No patients exhibited any predisposing factor for the development of mycosis, including systemic or immunosuppressive diseases, concomitant therapies, or an unbalanced diet.

Seven patients (15%) in the cohort developed candidiasis during BOT therapy.

**Fig. 1:**
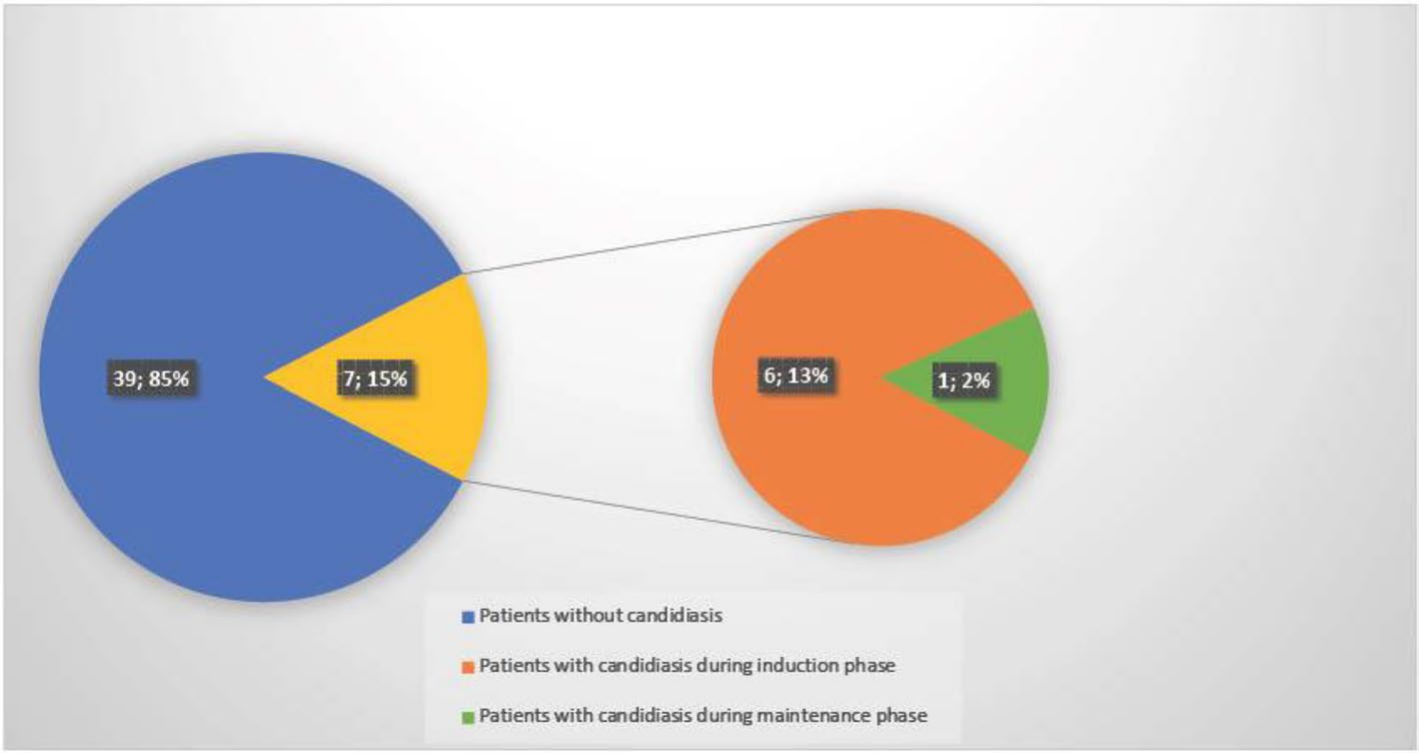
Distribution of patients: patients without candidiasis (39, 85%) are reported in blue; patients with candidiasis (7, 15%) are reported in yellow of which 6 (13%, in orange) during the induction phase and 1 (2%, in green) during the maintenance phase

Six patients (85%) were in the induction phase (1 mg twice a day), while one patient (15%) was in the early maintenance phase (0.5 mg twice a day) of the treatment (Fig. 1).

Five patients (71%) exhibited oral infection, while two patients (29%) demonstrated esophageal mycosis. During the induction phase (1 mg twice daily), three patients (42%) discontinued treatment due to recurrent (2, 67%) or severe oral candidiasis (1, 33%); the latter occurred subsequent to a prior episode of oral candidiasis. 

In terms of the characteristics of infection presentation, for patients affected by oral mycosis, the time of appearance after the commencement of BOT was approximately 10 weeks for them in the induction phase and about twenty weeks for the patient in the maintenance phase. In patients with esophageal involvement, candidiasis was identified during the initial gastroscopy conducted following the induction phase.

All patients presenting oral candidiasis exhibited symptoms characterized by a cotton-like sensation in the mouth and the presence of white plaques on the buccal mucosa, palate, and tongue. The infection persisted for approximately 10 days and was successfully treated with topical antifungal therapy (nystatin suspension 400,000 orally four times daily for 10 days).

All cases of esophageal candidiasis were asymptomatic and detected by endoscopy. The patients were treated with fluconazole (400 mg orally on day 1, followed by 200–400 mg daily for 14 days).

The treatments were efficacious in resolving the fungal infections in all patients.

### Endoscopic and histological features

Endoscopic features before treatment are summaries with a mean EREFS score of 2 for patients with and without candidiasis side effects.

After BOT induction phase, the mean value improved to 1.5 for the first group and to 1.3 for the second one.

Between patients with candidiasis, 2 of them (29%) improved this score and 5 (71%) remained as previous.

Concerning histological features, all the 7 patients with fungal infection presented peak eosinophil count (PEC) > 35/HPF before corticosteroid treatment, with mean count of 45 ± 8.6/HPF.

Patients without candida had a mean count of 33 ± 21/HPF.

All patients presented histologic remission after BOT induction treatment.

###  Concomitant therapies

All patients were not taking other gastrointestinal treatments nor systemic steroid therapies. Of the patients with candidiasis infection, 4 (57%) were in concomitant proton pump inhibitor therapy, all (100%) at 40 mg per day or more dosage (p<0.05). 3 patients of this group presented recurrent and severe oral candidiasis inducing discontinuation of treatment.

Of the patients without candidiasis infection, 15 (38%) were in concomitant PPI therapy, 6 (41%) at 40 mg per day or more dosage, and 9 (59%) at 20 mg per day.

### Recurrence

 2 of the 7 patients with mycosis (28%) presented recurrent oral candidiasis during induction phase inducing discontinuation of treatment.. They presented a peak eosinophil count ≥ 40/HPF at gastroscopy before treatment.

Two of them (67%) had concomitant treatment with PPI.

## Discussion

Eosinophilic esophagitis is a chronic immune-mediated disease characterized by eosinophil-predominant inflammation of the esophagus.

According to recent guidelines, topical steroids are effective for inducing histological and clinical remission during the induction and maintenance phases in the medium and long term [5, 8, 9, 15]. Other therapeutic options, like elimination diet and dupilumab, can be considered, but the first-line approach should be accurately evaluated according to patient features.

If compared to placebo, topical steroids present a good safety profile, without significant serious adverse events, as reported by several randomized controlled trials [10–12, 15, 16].

Data from a 6-week open-label trial of the EOS-2 Programme regarding BOT use report adverse drug reactions in 60 patients (33.1%) during therapy [7]. Only three of them experienced serious events, but not related to treatment. The most frequent adverse event was represented by infections and infestations (12.7%) with local candidiasis reported for 15 patients (8.3%). For all of them, intensity was mild or moderate and they recovered after local medical treatment. Esophageal candidiasis (EC) has been reported in up to 15% of patients undergoing topical steroid treatment [10–12, 15, 16]. 

In our study, we investigate risk factors for the development of oral and esophageal candidiasis during budesonide orodispersible tablets therapy in eosinophilic esophagitis patients.

Oral candidiasis is commonly caused by *Candida albicans*, a dysmorphic yeast that can present as both hyphal and yeast forms depending on the environment [20, 21].

*Candida* is part of the normal commensal oral microflora of immunocompetent individuals.

Risk factors for the pathologic colonization of *Candida* include age extremes (young children and elderly), metabolic diseases, immunocompromising conditions, concomitant infections, malnourishment, radiation therapy, organ transplantation, long-term steroid treatment, antibiotic treatment, and salivary gland hypofunction [22, 23].

Regarding EC, it has historically been associated with HIV (human immunodeficiency virus). Nevertheless, a recent study showed a decreasing prevalence of EC among patients with HIV. This was attributed to the rise in the use of highly active antiretroviral therapy. In addition, there was an increase in the prevalence of EC among patients without HIV [24]. In a recent multicenter retrospective case–control study, Kimchy et al. found that prior organ transplant, PPI, and corticosteroids were independent risk factors for the development of EC in patients without HIV [25]. Prior to this study, Ogiso et al. stated that for immunocompetent patients, PPI use, atrophic gastritis, advanced gastric cancer, and post-gastrectomy were critical risk factors for the development of EC [26].

The association between proton pump inhibitors and the development of esophageal candidiasis is still not well established. In a study of 2018, Nassar Y. et al. reported this association, explaining that this treatment may be associated with esophageal candidiasis in immunocompetent patients [27]. A potential mechanism leading to this infectious condition is represented by reduction of gastric acid secretion, potentially leading to a relative state of hypochlorhydria, changing the pH value in the stomach and inducing fungal disorders [28].

In addition, PPI can significantly alter gut microbiota [29], failing to restore dysbiosis of mycobiome and increasing colonization of *Candida* [30].

Furthermore, it has been reported that there is also a rare genetic component that predisposes to fungal infections and that has been identified in NADPH-oxidase malfunction [31–33] and abnormal production of some cytokines, such as the tumor necrosis factor α and interleukin 10 [34].

In our study, we included all patients in BOT therapy. Of these, we identified and compared two subgroups, one including 39 patients who did not develop candidiasis and one including patients with oral or esophageal fungal infection (7 patients, 15%), which was the only adverse event registered during the treatment. These data are slightly higher than that reported in the literature [7, 10, 18]. All these patients had no other risk factors for development of mycosis beyond steroid treatment.

Regarding concomitant therapies, all patients were not taking other gastrointestinal treatments nor systemic steroid therapies. The only concomitant therapy reported was PPI. In particular, of the patients with candidiasis infection, 57% were under this treatment, all of them (100%) at 40 mg per day or more dosage.  The subgroup that developed mycosis infection was on a statistically significative higher PPI dosage than the subgroup that did not develop this side effect (p<0.05, as shown in Table 1). On the contrary, a minor percentage of patients without mycosis (38%) was taking this medication. In this case, only 41% had a 40 mg per day or more dosage. As mentioned before, concomitant PPI therapy could represent a risk factor in developing mycosis in all patients, especially in those with EoE taking BOT therapy. According to literature [35, 36], the reasons are summarized in PPI-induced elimination of the gastric acid barrier and corticosteroid immunosuppression, both taking place in a major esophageal inflammation, leading to oral and esophageal candida colonization. In addition, a major PPI dosage, as in this case 40 mg per day or more, may play a role in predisposing the mycosis because of the increased efficacy and acid suppression obtained with the higher dose, as observed in the cohort with infection. The other group was taking this dose only in 41% of the total.

Another interesting result is the greater mean eosinophil count of 45 ± 8.6/HPF in patients with candidiasis infection than those without (33 ± 21/HPF). According to a post hoc analysis of an 8-week randomized trial by Cotton C.C. et al., the peak eosinophil count is not associated with severity of disease [37]. Nevertheless, the higher eosinophil count may reflect a major inflammatory weight, as also revealed macroscopically. Indeed, endoscopies performed after induction therapy showed a lesser improvement, although not statistically significant (p>0.05), in EREFS score in patients with fungal infection and 71% of them did not change the score, remaining as previous. This may induce us to suppose that a consistent eosinophilic count could be another risk factor in developing mycosis.

The limitations of this study are primarily associated with its retrospective design. In fact, it lacks longitudinal follow-up, which is useful to understand the progression and the recurrence of candidiasis infection after several months. Secondly, the limited sample size and its application to a single center may limit the generalizability of the findings to a broader population. Lastly, although clinical observation is generally sufficient to detect the presence of oral candidiasis, oral swabs were not obtained to confirm the diagnosis.

## Conclusions

Our results may suggest that concomitant PPI therapy and significant eosinophilic count could represent risk factors in developing mycosis in EoE patients. According to literature, major esophageal inflammation, PPI-induced elimination of the gastric acid barrier, and corticosteroid immunosuppression are the main mechanisms leading to oral and esophageal candida colonization. Concomitant use of PPI during BOT treatment in eosinophil esophagitis should be carefully evaluated, especially in patients with elevated eosinophil count. Further studies are needed in order to evaluate potential correlation and risk factors for the development of oral and esophageal candidiasis during budesonide therapy in EoE patients. 

## Data Availability

The data that support the findings of this study are available from the corresponding author upon reasonable request.
